# Persistent Coronary Vasomotor Tone During Myocardial Ischemia Occurs at the Capillary Level and May Involve Pericytes

**DOI:** 10.3389/fcvm.2022.930492

**Published:** 2022-06-24

**Authors:** D. Elizabeth Le, Yan Zhao, Sanjiv Kaul

**Affiliations:** ^1^Knight Cardiovascular Institute, Oregon Health & Science University, Portland, OR, United States; ^2^Cardiology Section, Department of Hospital and Specialty Medicine, Veterans Administration Portland Health Care System, Portland, OR, United States

**Keywords:** microvascular resistance, myocardial ischemia, capillaries, pericytes, vasodilators

## Abstract

**Background:**

There is persistent coronary vasomotor tone during myocardial ischemia, despite ongoing coronary arteriolar dilatation. The mechanism underlying this vasodilatory tone, which can be unmasked by coronary vasodilators, is unclear. We hypothesized that persistent microvascular resistance during myocardial ischemia occurs at the level of capillaries and may be caused by pericytes.

**Methods:**

We studied nine instrumented dogs where coronary blood flow and coronary driving pressure were reduced to half by placement of stenoses. Myocardial blood flow and myocardial blood volume were measured with myocardial contrast echocardiography before and during adenosine administration. In three animals, the heart was perfusion-fixed under these conditions for electron microscopic assessment of capillary and pericyte size.

**Results:**

During ischemia, myocardial blood volume decreased and myocardial vascular resistance remained unchanged. Adenosine administration reversed the decline in myocardial blood volume and decreased myocardial vascular resistance. Electron microscopy showed larger capillaries in ischemic beds receiving adenosine than ischemic beds not receiving adenosine. Pericytes in beds receiving adenosine also tended to be larger.

**Conclusion:**

Capillaries are the site of persistent vasomotor tone during myocardial ischemia; any other site of vascular regulation (arterioles or venules) cannot explain these myocardial contrast echocardiography findings, which are confirmed on post-mortem electron microscopic examination. The decrease in capillary size is likely caused by pericyte contraction in an attempt to maintain a constant capillary hydrostatic pressure. Adenosine relaxes pericytes, restores myocardial blood volume, reduces myocardial vascular resistance, and improves regional function during ischemia. These findings could have important therapeutic implications.

## Introduction

Several investigators have described the presence of residual coronary vasomotor tone during myocardial ischemia (1–5). This residual tone, which can be pharmacologically unmasked by the use of coronary vasodilators (1–9), is present, despite presumed exhaustion of arteriolar vasodilatory reserve when coronary blood flow (CBF) is reduced. The mechanism underlying this persistent vasomotor tone during myocardial ischemia has not been fully elucidated.

Using myocardial contrast echocardiography (MCE), we previously showed that when CBF decreases moderately at rest, myocardial blood volume (MBV), 90% of which resides in myocardial capillaries, decreases, while total microvascular resistance (MVR) remains unchanged (10). At very low coronary perfusion pressures, MVR actually increases (11, 12). For this study, we hypothesized that (a) either capillary constriction or decreased capillary density is the cause of the decline in MBV during myocardial ischemia, contributing to the residual vasomotor tone; and (b) this effect could be mediated by pericytes.

Although pericytes were described almost 150 years ago (13), differentiating them from vascular smooth muscle cells was difficult. Recent immunohistochemical techniques have made it easier to differentiate phenotypes of pericytes (14), and their ability to regulate local capillary blood flow under a wide range of conditions in several microvascular beds has been clearly demonstrated (15–17). Pericyte bodies decorate capillaries, and their processes surround capillaries and communicate with processes from other pericytes. Pericytes contract in response to pharmacological stimuli, including ATP and norepinephrine, and pericyte-mediated constriction and dilation of capillaries appear to modulate physiological microvascular blood flow regulation in the brain and eye (18–20).

We showed *in vivo* that when perfusion pressure decreases distal to a critical femoral artery stenosis during hyperemia, pericytes contract, resulting in capillary de-recruitment (21). This finding is attenuated in mice with partial pericyte depletion. More recently, we reported that during acute myocardial infarction where anterograde blood flow was reduced to zero, capillaries constricted at pericyte locations in order to maintain a constant capillary hydrostatic pressure (CHP), resulting in no reflow after reperfusion (22). We demonstrated that pericyte contraction was mediated by GPR39 and GPR39 knockout mice, and mice treated with a specific GPR39 inhibitor exhibited better capillary density, less no reflow, and smaller infarct sizes. Therefore, for the present study, we hypothesized that pericyte contraction could lead to capillary constriction, explaining the basis of persistent vasomotor tone during myocardial ischemia.

Here, in a canine model, where coronary physiology can be reliably assessed, we show that MBV increases and MVR decreases when adenosine is administered intracoronary during myocardial ischemia caused by reduced coronary driving pressure (CDP). Adenosine has been shown to relax pericytes by activating ATP-sensitive K+ channels (23–25). On electron microscopy (EM), ischemic myocardial beds infused with adenosine show larger capillary dimensions at similar CDP than ischemic beds not treated with adenosine. The purpose of this study was to determine the degree to which pericytes might participate in local blood flow regulation during myocardial ischemia.

## Methods

### Animal Preparation

The study was approved by the Animal Care and Use Committee at Oregon Health & Science University and conformed to the National Institutes of Health Guidelines for the Care and Use of Laboratory Animals. In total, nine adult male dogs (25–35 Kg) were studied. Female animals were not used to avoid potential for undetected pregnancy. The animals were intubated and ventilated with room air. Sodium pentobarbital was used to induce (40 mg.Kg^−1^) and maintain (200 mg.hr^−1^) anesthesia throughout the experiment. The heart rate, PO_2_, end-tidal CO_2_, and temperature were continuously monitored (Advisor® Vital Signs Monitor, Surgivet, Norwell, MA). Catheters (7F) were placed in both the femoral veins for microbubble infusion and administration of fluids and drugs, respectively.

**Figure 1:**
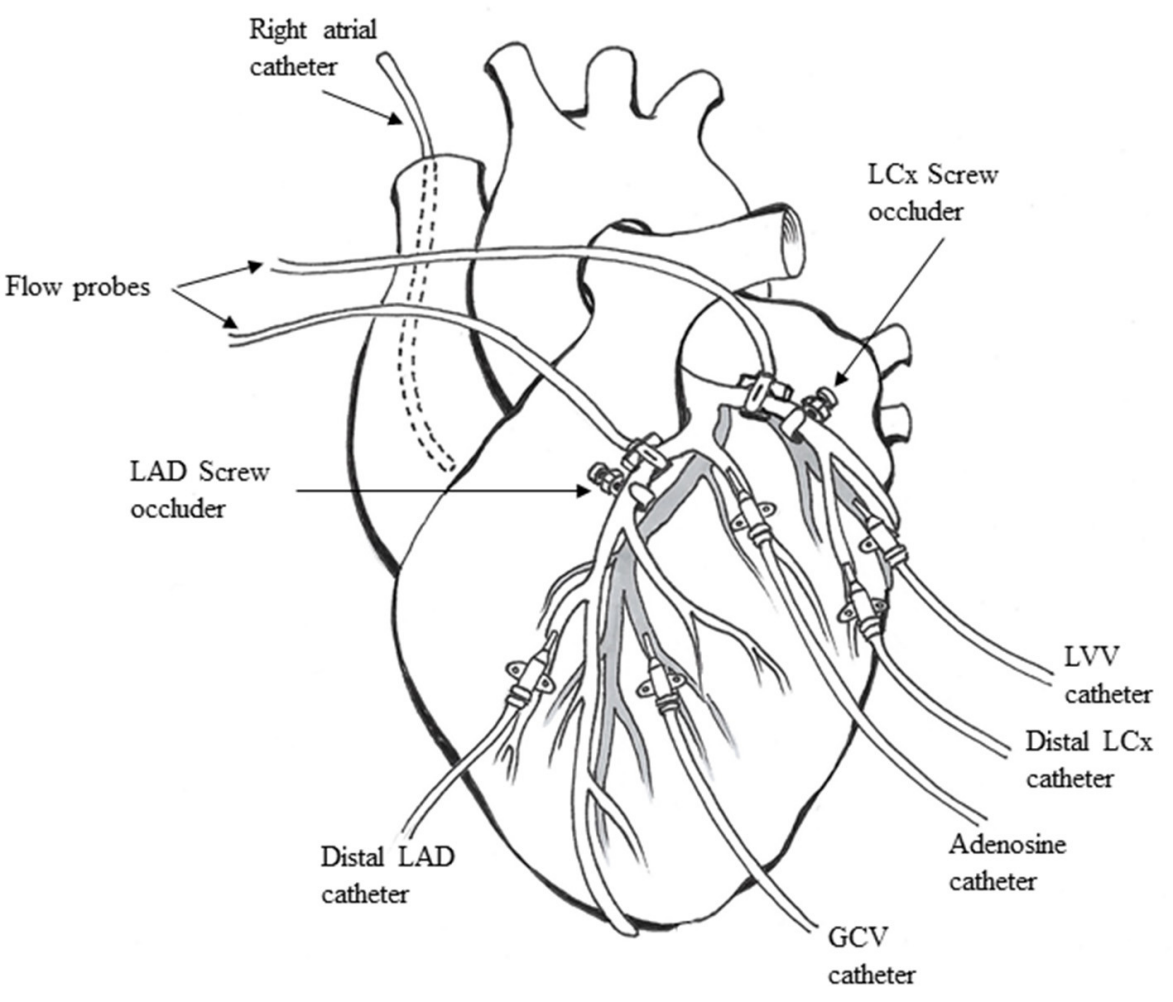
Animal preparation used for the experiments (see text for details). LAD, left anterior descending coronary artery; LCx, left circumflex coronary artery; GCV, great coronary vein; LVV, left ventricular vein.

A left lateral thoracotomy was performed, and the heart was suspended in a pericardial cradle. Catheters (7F) were inserted into the right atrium (RA) and retrogradely into the descending aorta to measure pressure. The proximal portions of the left anterior descending (LAD) and left circumflex (LCx) coronary arteries were dissected free from surrounding tissue. Time-of-flight ultrasonic flow probes (series SC, Transonics, Ithaca, NY) were placed on proximal portions of both arteries and connected to a digital flow meter (model T206, Transonics) to monitor and record CBF. A custom-designed occluder was placed on both coronary arteries distal to the flow probes (Figure 1). Polyethylene catheters (20 G) were inserted into the distal LAD and LCx to measure pressures. Either the LAD or LCx had an additional similar catheter placed through a side branch closer to the occluder to infuse adenosine. Polyethylene catheters (22 G) were placed in the great coronary vein adjacent to the LAD coronary artery and left ventricular vein parallel to the LCx coronary artery, respectively, to collect blood samples.

### Hemodynamic Measurements

Catheters and flow meters were interfaced to a multi-channel recorder (PowerLab, AD Instruments, Inc., Colorado Springs, CO). CBF and pressures in both coronary arteries, as well as mean central aortic and right atrial pressures, were acquired digitally and displayed on-line on a computer system (iMac, Apple Inc., Cupertino, CA). All data were saved and analyzed off-line using LabChart 6.

### Metabolite Measurements

Coronary venous blood lactate and pH were measured in the blood samples (0.8 mL in 1.0 mL heparinized syringes) obtained from both coronary veins. The samples were immediately placed on ice and analyzed within 15 min using an ABL800-FLEX analyzer (Radiometer Medical, Bronshoj, Denmark).

### Echocardiography

Two-dimensional echocardiography was performed using a Sonos-5500 System (Philips Healthcare, Andover, MA) interfaced to a phased-array S-3 transducer capable of harmonic imaging. The transducer was affixed to the procedure table using a custom-designed arm. A saline bath acted as an interface between the transducer and the heart. Ultrasound gel was placed between the bath and the anterior cardiac surface. For regional wall thickening (WT) measurements, real-time images were acquired at the mid-papillary muscle short-axis level and stored for later analysis.

Our method for WT quantification has been previously described (26). Briefly, the epicardial junction of the right ventricular posterior wall and the left ventricular free wall was identified, from which WT measurement was initiated in each frame, allowing for registration of the same endocardial and epicardial points between frames despite cardiac rotation around its long axis. Respiration-induced cardiac translation was avoided by briefly stopping the ventilator. A dozen targets were outlined on the endocardium and epicardium, respectively, in each frame from end-diastole to end-systole, which were then automatically connected using cubic spline interpolation. In total, 100 equidistant points were automatically defined on the epicardial outline to which a tangent was drawn and a line perpendicular to the tangent intersected the epicardium and endocardium as the shortest distance between them. The resulting chord lengths were measured in all frames from end-diastole to end-systole, with the first chord being the reference point. Using overlays derived from MCE images (see below), WT was averaged in the central 50% of the LAD and LCx beds and expressed as maximal percent change over the cardiac cycle.

For MCE, imaging was performed at the same mid-papillary short-axis level during a continuous infusion of diluted Lumason^®^ (10 mL mixed with 100 mL of normal saline, Bracco Diagnostics, Princeton, NJ) infused at 150 mL·hr^−1^. After steady state is achieved, this concentration of microbubbles produces adequate myocardial opacification without posterior wall attenuation. Images were acquired with ultrasound gated to the electrocardiogram at end-systole at pulsing intervals (PIs) of 1–3, 5, 8, 10, and 20 cardiac cycles to allow for progressively greater bubble replenishment of the ultrasound beam elevation (27). At least five images were obtained and digitally stored at baseline (pre-contrast) and at each PI during temporary suspension of ventilation.

Custom-designed software was used for image analysis (28). Pulsing interval vs. background-subtracted acoustic density plots were generated from regions of interest (ROI) placed on the LAD and LCx beds (defined on MCE by injection of diluted microbubble solution into each artery at beginning of the experiment, see below). They were fitted to an exponential function, y = *A* (1–e ^−β*t*^), where y = acoustic density at PI t, *A* = acoustic density after the ultrasound beam is completely replenished (MBV fraction), and β = rate constant that reflects the mean myocardial blood flow velocity. The product, *A*•β, was calculated to derive regional MBF (26, 27). Because the ultrasound signal is log-compressed for visualization, we converted all signals to a linear (0–255) scale using an anti-log function (29). Our MCE method of measuring MBF has been validated against the gold standard techniques of radioactive microspheres (27) and positron emission tomography (30). Because of inter-dog differences in ultrasound acoustics, all measurements were expressed as percent change from baseline. The inter- and intra-observer errors for MCE measurements from our laboratory have been previously published (31).

### Electron Microscopy

The heart tissue from each vascular bed was separated into endocardial, mid-wall, and epicardial one-thirds. Each section was then divided into multiple pieces measuring about 1 mm^3^ in size and fixed in 2.5% glutaraldehyde and 2.5% formaldehyde in 0.1 M sodium cacodylate buffer, pH 7.4, and processed in a PELCO Biowave Pro+ microwave with SteadyTemp Pro (Ted Pella, Redding, CA). The samples were rinsed three times with sodium cacodylate buffer for 40 s at 150 W, stained with 2% osmium tetroxide and 1.5% potassium ferricyanide for 13 min with a cycle of 3 min on at 100 W and 2 min off, rinsed with water three times for 40 s at 150 W, stained *en bloc* with uranyl acetate for 6 min at 100 W with a cycle of 2 min on, 2 min off, dehydrated in an acetone series for 40 s at 150 W, infiltrated with resin for 15 min at 150 W, and polymerized at 60 °C. The sections, 70 nm thick, were post-stained with uranyl acetate and lead citrate and imaged at 120 kV in an FEI Tecnai T12 (Thermo Fisher Scientific, Waltham, MA) transmission electron microscope equipped with a NanoSprint 12 (AMT Imaging, Woburn, MA) camera. Images of the heart were used to quantify the capillary circumference and diameter (averaged from two orthogonal planes in short axis views and multiple locations in long axis views). The size of pericytes and their processes surrounding the capillaries was also planimetered.

### Experimental Protocol

All baseline hemodynamic, CBF, 2-dimensional echocardiography, and MCE data were first obtained, followed by blood collection from veins. This sequence of data collection was repeated at each stage of the experiment. CBF and CDP for each artery were initially measured after 30 s of total coronary occlusion to define maximal hyperemic CBF. The LAD and LCx perfusion bed sizes were then defined *in vivo* using 0.1 mL of Lumason^®^ diluted to 3 mL with normal saline and injected sub-selectively into either artery. A primary stenosis was created on either an LAD or LCx to reduce CBF to below 50% of baseline, and all data were collected. Adenosine was then infused into the artery (5 μg·kg^−1^·min^−1^ at a rate of 3 mL·min^−1^). Four min into infusion, data collection was initiated after completion of which adenosine infusion was discontinued. A secondary flow reducing stenosis was then placed on the other artery (positive control), and all data were collected again.

After completion of the aforementioned stages, adenosine infusion was restarted in the artery that had previously received it. In three dogs, a mixture of 2% glutaraldehyde and 2% paraformaldehyde in 0.1 M phosphate buffer, pH 7.2, was infused in both arteries at the same rate for EM studies. After 10 min of infusion, the animals were euthanized with a mixture of pentobarbital and KCl, and the hearts were removed and cut into 5 short-axis slices. The slice corresponding to the MCE images (defined by placing a needle through the heart at the level of the ultrasound transducer) was processed for EM. The LAD and LCx beds were defined based on MCE images obtained during intracoronary injection of microbubbles.

### Statistical Methods

Data are expressed as mean ± 1 SEM. Comparisons between stages were performed using either ANOVA or unpaired Student's *t*-test. A modified Bonferroni correction was used for multiple comparisons. For differences between groups, a *p*-value of <0.05 (two-sided) was considered significant.

## Results

CBF at rest and maximal hyperemia (mean ± 1SEM) were 35 ± 4 mL·min^−1^ and 94 ± 13 mL·min^−1^ for the LAD and 36 ± 4 mL·min^−1^ and 79 ± 13 mL·min^−1^ for the LCx, respectively. Primary stenoses (those examined before and after adenosine) were created in nine animals. One animal died after creation of the primary stenosis; hence, secondary stenoses were created in eight animals.

### Hemodynamic Results

Table 1 lists the hemodynamic results pertaining to the primary stenosis before and after adenosine administration. By design, CBF was reduced to half by stenosis creation, resulting in marked reduction in distal CDP. The heart rate showed a significant decline compared to baseline but mean aortic pressure, and the rate pressure product remained unchanged. As noted by us previously (11, 12), despite arteriolar dilatation, total MVR (distal coronary pressure-RA pressure/CBF) did not change at this level of stenosis. %WT declined by the same magnitude (53–66%) as CBF. The presence of ischemia was corroborated by a significant decline in coronary venous pH and increase in coronary venous lactate levels. Comparable results were noted when the secondary stenosis was created (Table 2), although the increase in coronary venous lactate and pH did not reach statistical significance presumably because of the smaller number (*n* = 5) of complete blood collections. Importantly, the distal coronary pressure was similar after creation of both stenoses.

**Table 1:** Primary stenosis results (*n* = 9, mean ± 1SEM).

**Variable**	**Baseline**	**Primary** **stenosis**	**Primary stenosis +** **adenosine**	**Anova**
Heart rate (bpm)	100 ± 7	90 ± 5^*^	94 ± 5	0.46
Systolic aortic pressure (mmHg)	129 ± 6	121 ± 9	125 ± 8	0.76
Mean aortic pressure (mmHg)	100 ± 4	99 ± 3	96 ± 4	0.90
RPP x 1,000 [beats·(min·mmHg)^−1^]	12.6 ± 0.9	11.5 ± 0.9	11.5 ± 0.8	0.56
Right atrial pressure (mmHg)	8 ± 1	10 ± 2	10 ± 2	0.65
Coronary blood flow (mL·min^−1^)	36 ± 5	17 ± 3^*^	23 ± 4	0.015
Distal coronary pressure (mmHg)	97 ± 4	43 ± 5^†^	37 ± 4^‡^	<0.0001
Coronary driving pressure (mmHg)	88 ± 4	36 ± 6^†^	29 ± 5^†^	<0.0001
MVR [mmHg·(mL·min)^−1^]	3.07 ± 0.57	2.89 ± 0.84	1.82 ± 0.66	0.41
% Wall thickening	38.2 ± 0.4	15.3 ± 2.7^*^	17.8 ± 1.4^†^	<0.0001
Coronary venous pH	7.29 ± 0.03	7.16 ± 0.04^*^	7.21 ± 0.03^‡^	0.05
Coronary venous lactate (mmol·L^−1^)	0.61 ± 0.03	1.94 ± 0.51^*^	1.73 ± 0.42^*^^‡^	0.05

*RPP, rate pressure product; MVR, myocardial vascular resistance.*

* *p < 0.05 vs. baseline.*

† *p < 0.001 vs. baseline.*

‡ *p < 0.05 vs. primary stenosis*.

**Table 2:** Secondary stenosis results (mean ± 1SEM).

**Variable**	**Baseline** ***n*= 8**	**Secondary** **stenosis** ***n*= 8**
Heart rate (bpm)	96 ± 7	85 ± 4^*^
Systolic aortic pressure (mmHg)	129 ± 6	120 ± 10
Mean aortic pressure (mmHg)	103 ± 5	95 ± 4
RPP x 1,000 [beats·(min·mmHg)^−1^]	12.1 ± 0.9	10.3 ± 0.8
Right atrial pressure (mmHg)	8 ± 1	10 ± 2
Coronary blood flow (mL·min^−1^)	39 ± 4	15 ± 3^†^
Distal coronary pressure (mmHg)	96 ± 4	43 ± 5^†^
Coronary driving pressure (mmHg)	87 ± 4	34 ± 5^†^
% Wall thickening	38.4 ± 0.3	17.5 ± 3.6^†^
	***n*** **=** **5**	***n*** **=** **5**
Coronary venous pH	7.29 ± 0.01	7.22 ± 0.03
Coronary venous lactate (mmol·L^−1^)	0.70 ± 0.03	1.56 ± 0.45

*RPP, rate pressure product; MVR, myocardial vascular resistance.*

* *p < 0.05.*

† *p < 0.001*.

During adenosine infusion into the coronary artery with the primary stenosis heart rate, mean aortic pressure and the rate pressure product remained unchanged (Table 1). CBF increased without reaching statistical significance, but the distal coronary pressure decreased significantly with a concomitant reduction in MVR, indicating persistent microvascular reserve despite a flow-reducing stenosis. %WT increased in line with the increase in CBF, and there was a significant increase in coronary venous pH and a decrease in coronary venous lactate, indicating partial amelioration of ischemia from adenosine administration.

**Figure 2:**
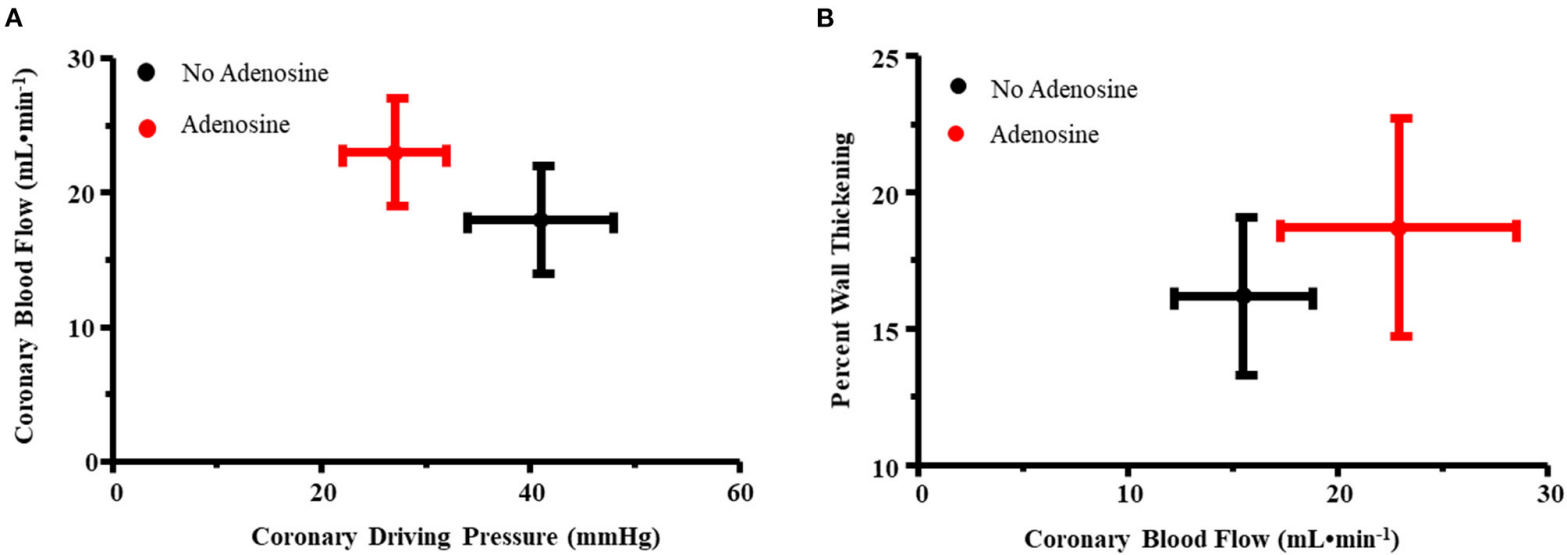
**(A)** Relation between coronary driving pressure (difference between distal coronary and RA pressures, x-axis) and coronary blood flow (y-axis) for the primary stenosis in all nine dogs prior to and during adenosine administration. An upward and leftward shift was noted with adenosine, indicating a reduction in myocardial vascular resistance. **(B)** Relation between coronary blood flow (x-axis) and percent wall thickening (y-axis) before and during adenosine administration. An upward and rightward shift occurred, indicating an increase in percent wall thickening during adenosine administration resulting from an increase in coronary blood flow.

Figure 2A illustrates the relation between coronary driving pressure (difference between distal coronary and RA pressures) and CBF for the primary stenosis in all nine dogs prior to and during adenosine administration. An upward and leftward shift was noted with adenosine, indicating a reduction in MVR. Figure 2B illustrates the relation between CBF and %WT before and during adenosine administration. An upward and rightward shift occurred, indicating an increase in %WT during adenosine administration resulting from an increase in CBF.

### MCE Results

Table 3 shows the results for the myocardial regions supplied by the primary and secondary stenoses expressed as percent change from baseline in six dogs where complete MCE data were obtained. Without adenosine, as expected, MBV (*A*) decreased after placement of both stenoses as did MBF velocity (β) and MCE-derived MBF. The decreases were quite similar for both the primary and secondary stenoses, confirming that the secondary stenosis was an adequate positive control. During adenosine infusion, MBV (*A*) in the bed supplied by the primary stenosis increased significantly, but MBF velocity (β) did not change. All the increase in MCE-derived MBF was therefore a consequence of the increase in MBV (*A*). There were no significant changes in MCE parameters in the bed supplied by the secondary stenosis (defined on MCE by injecting microbubbles in the artery where stenosis was subsequently placed) when adenosine was infused into the bed with primary stenosis (Table 4).

**Table 3:** Percent change in MCE variables compared to baseline values (*n* = 6, mean ± 1SEM).

	**Primary stenosis**		**Secondary stenosis**
**Variable**	**Before adenosine**	**During adenosine**	**(No adenosine)**
*A*	−39 ± 9	118 ± 40^*^	−33 ± 14
*B*	−45 ± 11	−25 ± 25	−46 ± 9
*A · β* (MCE-derived MBF)	−63 ± 11	97 ± 59^*^	−63 ± 12

*MCE, myocardial contrast echocardiography; MBF, myocardial blood flow.*

* *p < 0.05 vs. baseline—primary stenosis before adenosine*.

**Table 4:** MCE variables from secondary stenosis region before and during adenosine infusion into the primary stenosis bed (*n* = 8, mean ± 1SEM).

**Variable**	**Before adenosine**	**During adenosine**
*A*	2.81 ± 0.95	2.40 ± 0.61
β	0.28 ± 0.06	0.24 ± 0.04
*A ·β* (MCE-derived MBF)	0.66 ± 0.17	0.49 ± 0.11

*MCE, myocardial contrast echocardiography; MBF, myocardial blood flow*.

### EM Results

**Figure 3:**
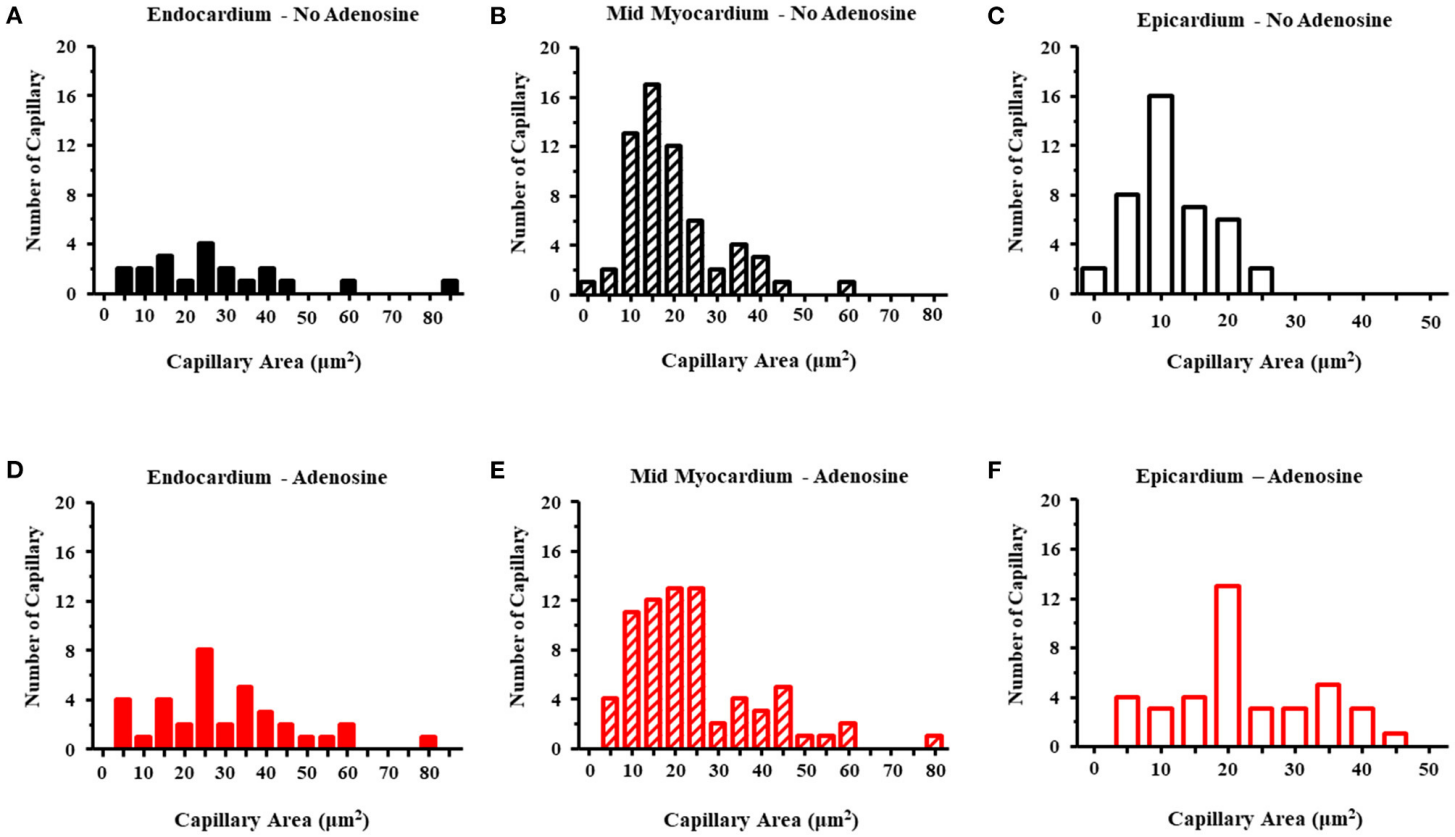
Histograms of the capillary area without adenosine in the endocardium **(A)**, mid-myocardium **(B)**, and epicardium **(C)** and with adenosine in the endocardium **(D)**, mid-myocardium **(E)**, and epicardium **(F)**. The capillary sizes are depicted on the x-axis in increments 5 μm^2^. The frequency of capillary measurements is depicted on the y-axis.

Table 5 lists the results from the three dogs from which EM results were obtained. For the most part, EM images depicted cross-sectional capillary planes. A total of 291 capillary areas were measured from the epicardium, mid-myocardium, and endocardium. Significant differences were noted in the capillary size between the different myocardial layers in beds in the absence of adenosine, with the largest capillaries seen in the endocardium and the smallest in the epicardium. The endocardial capillary area was approximately 45% larger than the mid-myocardial area, which in turn was approximately 70% larger than the epicardial area. The transmural differences in the capillary size disappeared in the beds in the presence of adenosine. Overall, the capillary areas were significantly larger in myocardial beds receiving adenosine than those not receiving adenosine in the same animals, despite similar severity of stenosis in the coronary arteries supplying these beds. In some instances, capillaries were observed in the longitudinal plane: in the 28 such instances, the capillary diameters were significantly larger in beds receiving adenosine than those not receiving adenosine. The histograms in Figure 3 depicts the distribution of capillary area in each myocardial layer in the absence and presence of adenosine. The distribution of all sizes remained the same in the endocardium and mid-myocardium before and during adenosine. In contrast, there was a marked shift to the right in the epicardium after adenosine, indicating an increase in capillary size with adenosine.

**Table 5:** Electron microscopy results (mean ± 1SEM).

	**No adenosine**	**Adenosine**
**A. Capillary area (μm** ^**2**^ **)**		
Endocardium	28.34 ± 4.37 (*n* = 20)	29.76 ± 2.83 (*n* = 36)
Mid-myocardium	19.53 ± 1.34 (*n* = 62)^‡^	24.02 ± 1.71 (*n* = 72)*
Epicardium	11.49 ± 0.92 (*n* = 41)^§#^	22.57 ± 1.76 (*n* = 39)^†^
	^‡^p < 0.01 v. endocardium ^§^p < 0.001 v. endocardium ^#^p < 0.01 v. mid-myocardium	ANOVA = 0.07 *p < 0.05 v. no adenosine ^†^p < 0.001 v. no adenosine
**B. Longitudinal capillary diameter (μm)**		
Anywhere in entire myocardial thickness	2.74 ± 0.19 (*n* = 10)	4.43 ± 0.76 (*n* = 18)*
		*p < 0.05 v. no adenosine
**C. Pericyte area surrounding capillaries (μm** ^**2**^ **)**		
Endocardium	0.89 ± 0.19 (*n* = 30)	1.22 ± 0.22 (*n* = 27)
Mid-myocardium	0.97 ± 0.10 (*n* = 48)	0.90 ± 0.11 (*n* = 58)
Epicardium	1.29 ± 0.21 (*n* = 32)	1.09 ± 0.21 (*n* = 32)

Measurements of pericyte areas were made where pericytes surrounded capillaries as not all capillary cross-sections were associated with pericytes. The size of the pericytes surrounding the capillaries tended to be larger in the endocardium and epicardium of beds receiving adenosine. The size includes both pericyte bodies and processes. The latter were more abundant. This difference did not reach statistical significance.

**Figure 4:**
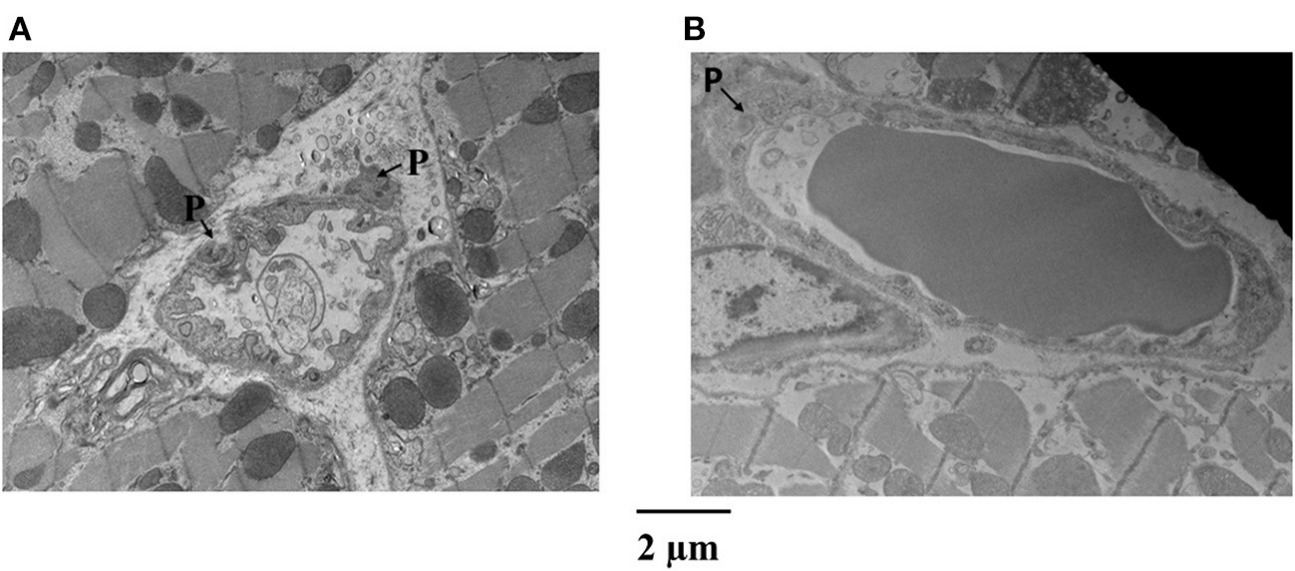
Examples of capillaries and their relation with pericytes (P) from the epicardium of a dog where CDP dropped from 42 to 34 mmHg and MVR decreased in that bed from 1.14 to 0.94 mmHg (mL·min)^−1^ when adenosine was infused. **(A)** Shows a capillary from the bed not receiving adenosine, while **(B)** depicts a capillary from the bed receiving adenosine. The capillary in **(A)** is surrounded by several pericytes and demonstrates many luminal invaginations. Not only the capillary diameter is smaller in **(A)** than in **(B)** but also the luminal surface is highly irregular.

Figure 4 is an example of capillaries and their relation with pericytes from the epicardium of one dog, where CDP dropped from 42 to 34 mmHg and MVR decreased from 1.14 to 0.94 mmHg (mL·min)^−1^ when adenosine was infused. Panel A shows a capillary from the bed not receiving adenosine, while panel B depicts a capillary from the bed receiving adenosine. The capillary in panel A is surrounded by several pericytes and demonstrates many luminal invaginations presumably caused by pericyte processes as previously described (32). Thus, not only the capillary diameter is smaller in panel A than in panel B but also the luminal surface is highly irregular. Such capillaries with multiple invaginations were more likely to be seen in the epicardium vs. other myocardial layers.

**Figure 5:**
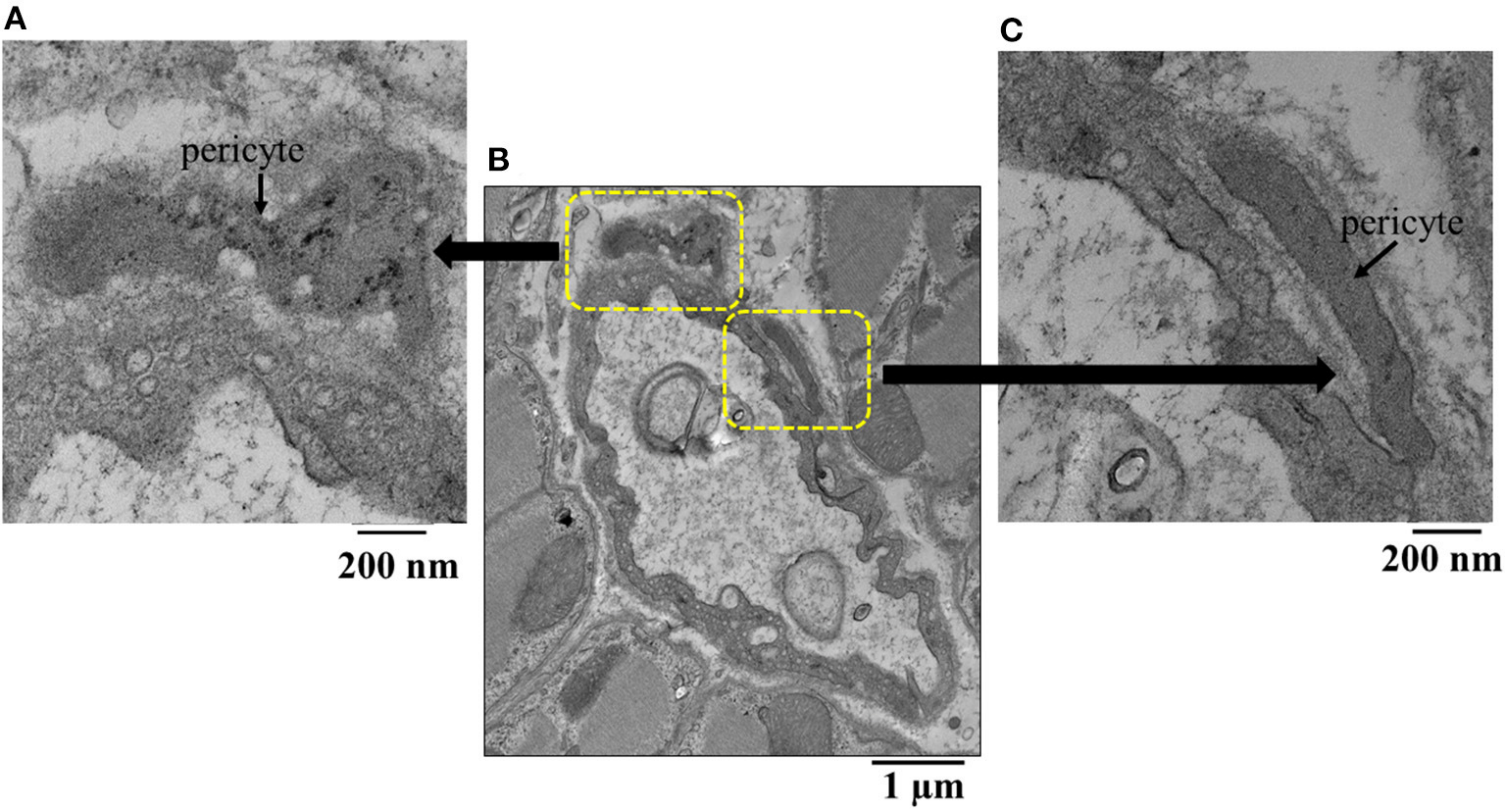
**(B)** Example of an epicardial capillary with multiple invaginations and surrounded by pericytes shown in high magnification inserts **(A,C)**.

Figure 5 illustrates another example of a capillary (middle panel) from the epicardium showing a shriveled appearance. Here, magnifications of original regions are also depicted (left and right panels), showing pericytes co-located at different parts of the capillary. The epicardium seemed to show the greatest difference in capillary areas between beds receiving and not receiving adenosine compared to other beds where the differences did not reach statistical significance (Table 5).

**Figure 6:**
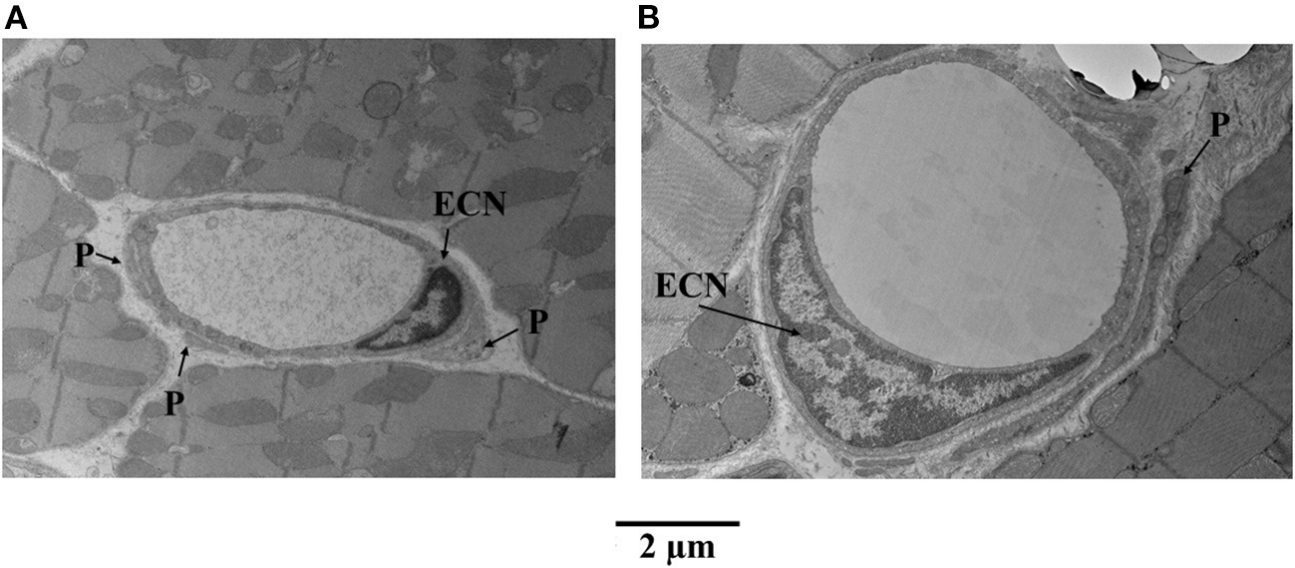
Examples of capillaries and pericytes (P) from the mid-myocardium of a dog, where MVR decreased even more during adenosine infusion [2.45 to 0.69 mmHg·(mL·min)^−1^]. **(A)** Shows a capillary from a bed not receiving adenosine, and **(B)** shows a capillary from a bed receiving adenosine. The capillary area is larger in the latter **(B)**. In both examples, endothelial cell nuclei (ECN) are also present as are pericyte processes.

Figure 6 illustrates examples of capillaries and pericytes from the mid-myocardium of another dog, where MVR decreased even more during adenosine infusion [2.45 to 0.69 mmHg·(mL·min)^−1^]. Here again, panel A shows a capillary from a bed not receiving adenosine, and panel B shows a capillary from a bed receiving adenosine. The capillary area is larger in the latter. In both examples, endothelial cell nuclei are also present as are pericyte processes.

**Figure 7:**
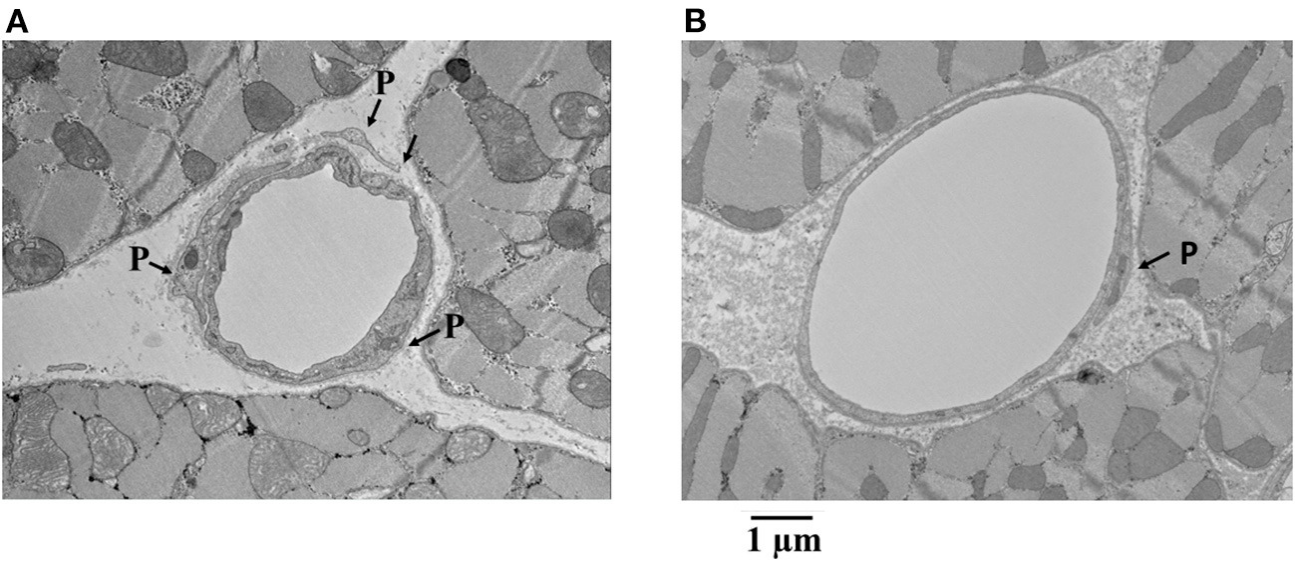
Examples of capillaries and pericytes (P) from the endocardium of a dog where MVR decreased from 2.58 to 1.28 mmHg · (mL · min)^−1^ and CDP decreased from 55 to 40 mmHg with adenosine infusion. Note the irregular appearing smaller lumen in **(A)** where adenosine was not given compared to the smoother and larger lumen where adenosine was given **(B)**. In both instances, pericyte processes are seen.

Figure 7 shows examples of capillaries and pericytes from the endocardium of yet another dog. Here, the MVR decreased from 2.58 to 1.28, and CDP fell from 55 to 40 mmHg with adenosine infusion. Note the irregular appearing smaller lumen in panel A was not given adenosine, whereas the smoother and larger lumen was given adenosine in panel B. In both instances, pericyte processes are seen.

## Discussion

The novel finding of this study is that a decrease in MBV (measured *in vivo* using MCE) during reduced CDP underlies the persistent microvascular tone seen during myocardial ischemia, since it reverses with adenosine administration. Thus, capillaries, which comprise 90% of MBV on MCE, are the site of persistent microvascular tone during myocardial ischemia; any other site of vascular regulation (arterioles or venules) cannot explain these findings because arterioles and venules contribute minimally to MBV measured on MCE during systole (27). These findings are confirmed on post-mortem EM examination of capillary size. This persistent microvascular tone during myocardial ischemia could be caused by pericyte contraction in an attempt to maintain a constant CHP. Adenosine relaxes pericytes, restores MBV, reduces MVR, and improves regional function during ischemia.

We previously found that when CBF decreases at rest, MBV as assessed by MCE decreases, while counter-intuitively MVR (CDP/CBF) remains the same or even increases (10–12): the lower the CDP, the lower the MBV and the higher the MVR (11, 12). If there were no countervailing forces increasing MVR during myocardial ischemia, arteriolar dilation alone should decrease MVR progressively with increasing stenosis severity, despite segmental differences in the degree of arteriolar dilation. Although MVR was not directly measured or calculated in previous studies describing persistent microvascular tone during ischemia, we were able calculate MVR from data presented by Pantely et al. (4) and Grattan and co-workers (5). In both instances, MVR increased with decreasing CDP below the autoregulatory range. The reason for this graded increase in MVR with lowering of CDP is unknown, and at the time of our previous studies, we postulated that “pre-capillary sphincters” could be responsible for this finding (10, 11). These pre-capillary sphincters are now considered likely to comprise pericytes (33).

Tissue homeostasis is essential for cell health and is maintained by the hydrostatic and oncotic pressures in the capillary and interstitium, respectively, as described by Starling 125 years ago (34). Of these forces, the most likely to fluctuate acutely is the CHP because of changes in systemic pressure. In the heart, the resistance arterioles maintain a constant CHP when the mean CDP ranges from approximately 45 mmHg to 120 mmHg. Below 45 mmHg aortic pressure, it is presumed that the arterioles fully dilate and cannot dilate anymore (35, 36).

Using two-photon microscopy in an *in vivo* neural/glial antigen 2 mouse hind limb preparation where pericytes appear red, we recently demonstrated that when perfusion pressure drops beyond a critical femoral artery stenosis during exogenous hyperemia, capillaries constrict at site of pericyte locations, and this finding is significantly attenuated in mice with partial pericyte depletion (21). More recently, we demonstrated the same phenomenon post-mortem in an acute myocardial infarction model with mice undergoing coronary occlusion, followed by reperfusion. Capillary constriction at the pericyte location was associated with the no reflow phenomenon (22). The inhibition of pericyte contraction either through gene knockout or drug treatment resulted in less capillary constriction and no reflow, as well as smaller infarct size. Taken together, our results imply a role of pericytes in the increased vasomotor tone noted during myocardial ischemia. For the current study, we, therefore, hypothesized that the persistent vasomotor tone and unchanged MVR noted during resting ischemia result from capillary constriction and/or reduction in capillary density caused by pericyte contraction in response to reduced CDP.

The dog myocardial mean capillary diameter is 5.5 μm when the heart is fixed at normal perfusion pressure (37, 38). In our study, we found that capillary size was reduced to half this size when CBF and CDP were reduced by half and increased with adenosine infusion to about 80% of the normal size, despite a further drop in CDP caused by adenosine. Consequently, the decrease in capillary size during myocardial ischemia cannot be simply a passive response to decreased intraluminal pressure. Reduction in CDP was associated not only with smaller capillaries but also with irregular shapes ones. These irregular shaped capillaries are more likely to increase MVR for the same capillary diameter because they impede the red cell transit.

It is not clear what happens to pericyte size when they contract. In most *in vivo* studies, pericyte contraction is implied by a change in capillary dimensions at the site of pericytes. *In vitro* studies have shown that pericyte bodies get smaller, rather than larger, on stimulation (39) and cramp the matrix on which they are placed (40). Simulation studies suggest that a change in the pericyte body shape itself can buckle the underlying capillary basement membrane (41). If the pericyte processes extend out in order to “dig into” the capillary, they will become thinner and longer. We could not find a single study where dimensions of pericyte bodies or processes were made *in vivo* during pericyte contraction.

There could be other potential reasons for increased microvascular tone during ischemia. For instance, increased α2-adrenergic activity during ischemia, particularly in anesthetized dogs, could increase microvascular resistance (42, 43). At the same time, α-1 adrenergic activity is required for the vasodilatory effect of adenosine released during ischemia (44). In any case, because arteriolar blood volume constitutes only a small fraction of MBV (45, 46), it is unlikely that MBV would be reduced from any arteriolar vasoconstriction that could result from sympathetic overdrive during myocardial ischemia.

Chilian et al. (2) showed that at lower CDP, adenosine caused dilatation of smaller arterioles that had not dilated fully during ischemia and suggested incomplete small arteriolar dilatation as a probable cause of persistent vasomotor reserve during myocardial ischemia. Based on our findings, we believe that the dilatation of smaller arterioles may have occurred as a consequence of increased distal flow caused by reduced resistance in the capillary network resulting from pericyte relaxation and capillary dilatation (47). The same authors also reported that coronary venous pressure increased during vasodilation, which passively increases capillary pressure (48). However, they did not measure capillary pressure directly and extrapolated curves generated from arteriolar and venous data (49). The predicted increase in CHP in their study required that capillary dimension, and hence, MBV remained unchanged during dipyridamole-induced vasodilation. If the capillary size increases as shown by us, CHP in their study would have remained the same before and after vasodilation.

Another explanation may be the vascular waterfall mechanism postulated by Downey and Kirk (50, 51), which implicates different intramyocardial pressures for the differences in systolic flow between various myocardial regions. They suggested vascular collapse during systole as the cause of the waterfall phenomenon and conjectured coronary veins to be the site of vascular collapse. Thus, higher endocardial intramyocardial pressures should cause greater coronary venous collapse that could retrogradely increase capillary pressure and hence capillary size in the endocardium compared to the mid-myocardium and epicardium as noted in the beds not receiving adenosine in our study. In our model, ischemia occurred in both myocardial beds, and the capillary dimensions were definitely larger in the endocardium with or without adenosine, which suggests that pericytes may not have been able to modulate capillary size under these conditions.

Whereas the gradation of capillary sizes across the different myocardial regions in beds not receiving adenosine would support the waterfall hypothesis, the increase in capillary sizes in the epicardium and mid-myocardium with adenosine would indicate higher mid-myocardial and epicardial tissue pressure caused by adenosine, resulting in larger capillaries. It is known that adenosine increases intramyocardial pressure through the Greg phenomenon (52).

We did not measure capillary size in the normal myocardium in our own study because this would have required perfusion fixation of another coronary artery at normal pressure, which was technically not possible. Reduction in CDP was not only associated with smaller capillaries but with irregular shaped ones. These irregular shaped capillaries are more likely to further increase MVR for the same mean capillary diameter by impeding erythrocyte transit.

### Study Limitations

The notion that pericytes contract to regulate CHP and hence MBF is novel and likely to be controversial. To that end, we would have liked to demonstrate the phenomenon *in vivo*, which, unlike the skeletal muscle, is not possible in the beating heart. Our examination of capillary dimensions and their relation to pericytes was performed post-mortem, where several factors can lead to measurement errors. We tried to mitigate this by perfusion fixing the heart at CDPs and CBFs that were similar to those when *in vivo* MCE measurements were made. Rather than using an additional group of dogs as controls, we used a different coronary artery to create comparable stenosis where adenosine was not administered.

A second limitation is that without three-dimensional information, we cannot be certain that the measured capillary areas represented the true cross-sectional areas that are 90 degrees from the longitudinal axis. Furthermore, although we measured the capillary size, EM does not allow the assessment of capillary density. As shown by us previously *in vivo*, pericyte contraction during a drop in perfusion pressure is associated with a decrease in both capillary dimension and density (21, 22). We have previously reported no difference in endocardial vs. epicardial MBV during myocardial ischemia (53). In this situation, a reduction in endocardial myocardial blood flow velocity was the basis of reversal of the endocardial/epicardial MBF ratio. Since MBV did not change in the endocardium during ischemia, an increase in capillary size would necessarily be associated with a decrease in capillary density. A decrease in capillary density can occur when entire capillary beds are closed off by pre-capillary sphincters that are also composed of pericytes (33, 54, 55). A combination of capillary de-recruitment and a decrease in dimension would be effective means for controlling CHP. The third limitation of the pericyte size analysis is that two-dimensional area measurement is subject to sampling variation. The pericyte areas that we measured included both cell bodies and the primary and secondary processes with unknown orientation in their relation to the capillaries.

Finally, we could not compare capillary size during both normal and reduced CDPs because of our inability to perfuse the same coronary bed before and after occlusion since immediate tissue harvest was obligatory for EM analysis. These anatomic data would have allowed us to determine if there was a comparable change to the MCE-derived MBV and MBF data.

### Clinical Implications

Unlike Pantely et al. (4) and similar to Aversano and colleagues (3), we found that adenosine administration caused improvement in regional function, despite persistent ischemia, which mirrored the increase in MBF caused by adenosine. Thus, pharmacologically addressing persistent vasomotor tone during myocardial ischemia could be of potential clinical benefit by improving regional myocardial function, which, in turn, could favorably impact global left ventricular function, especially if multiple myocardial beds are ischemic. Even after coronary bypass surgery, a significant proportion of patients continue to experience angina. In many instances, there are smaller coronary artery branches that have critical or worse stenosis not amenable to bypass (56).

Our results also shed new light on some of our own previous observations. For instance, we recently showed that ranolazine increases tissue adenosine levels by directly increasing cytosolic-5'-nucleotidase activity (57). Although there are several possible pathways of angina relief by ranolazine, pericyte relaxation in response to increased tissue adenosine levels could be an additional mechanism. We also showed that nitroglycerin has beneficial effects on the microcirculation by increasing S-nitrosohemoglobin levels, which causes greater oxygen unloading from erythrocytes during ischemia (58). However, nitric oxide can also relax pericytes by inhibiting calcium and chloride currents (15, 59), which may have been an additional mechanism of increased tissue MBF and ischemia relief by nitroglycerin.

Other anti-anginal medications could also act by relaxing pericytes. For instance, nicorandil has the dual properties of a nitrate and ATP-sensitive K^+^ channel agonist (60), both of which can cause pericyte relaxation. Similarly, calcium channel blockers, such as verapamil, may also limit pericyte contraction by inhibiting L-type voltage-dependent calcium channels, as shown for nifedipine (61). None of these pharmacological maneuvers are specific to pericytes and may not completely block pericyte contraction. For instance, despite pharmacological doses of adenosine, capillaries did not achieve their normal size in our study. Thus, there may be room for additional pharmacological maneuvers aimed at completely inhibiting pericyte contraction.

## Summary and Conclusion

In summary, a decrease in MBV (measured *in vivo* using MCE) and an increase in MVR during reduced CDP underlie the persistent microvascular tone seen during myocardial ischemia, since these findings reverse with adenosine administration. These findings indicate that capillaries are the site of persistent microvascular tone during myocardial ischemia. Any other site of vascular regulation (arterioles or venules) cannot result in this combined effect of decreased MBV and increased MVR. These findings are confirmed on post-mortem EM examination of capillary size. We provide circumstantial evidence to indicate that pericyte contraction may be responsible for the reduction in MBV. Direct *in vivo* observations will be required to confirm these findings, which could have important clinical implications for the treatment of myocardial ischemia.

## Data Availability Statement

The raw data supporting the conclusions of this article will be made available by authors upon request, without undue reservation.

## Ethics Statement

The animal study was reviewed and approved by the Animal Care and Use Committee at Oregon Health & Science University.

## Author Contributions

DL developed and performed the experiments, analyzed and tabulated the results, and drafted the manuscript. YZ assisted with the experiments. SK developed the hypothesis and study design, assisted with the experiments, reviewed the data, and helped with manuscript development. All authors contributed to the article and approved the submitted version.

## Funding

This research was supported by the Garthe and Grace L. Brown Fund of the Oregon Community Foundation.

## Author Disclaimer

The contents do not represent the views of the U.S. Department of Veterans Affairs or the U.S. government.

## Conflict of Interest

The authors declare that the research was conducted in the absence of any commercial or financial relationships that could be construed as a potential conflict of interest.

## Publisher's Note

All claims expressed in this article are solely those of the authors and do not necessarily represent those of their affiliated organizations, or those of the publisher, the editors and the reviewers. Any product that may be evaluated in this article, or claim that may be made by its manufacturer, is not guaranteed or endorsed by the publisher.
